# Efficacy and Toxicity of Fexinidazole and Nifurtimox Plus Eflornithine in the Treatment of African Trypanosomiasis: A Systematic Review

**DOI:** 10.7759/cureus.16881

**Published:** 2021-08-04

**Authors:** Jessica Hidalgo, Juan Fernando Ortiz, Stephanie P Fabara, Ahmed Eissa-Garcés, Dinesh Reddy, Kristina D Collins, Raghavendra Tirupathi

**Affiliations:** 1 Internal Medicine, San Francisco de Quito University, Quito, ECU; 2 Neurology, Larkin Community Hospital, Miami, USA; 3 Internal Medicine, Santiago de Guayaquil Catholic University, Guayaquil, ECU; 4 Neurology, San Francisco de Quito University, Quito, ECU; 5 Neurology, Mysore Medical College, Mysore, IND; 6 Internal Medicine, University of the West Indies, Kingston, JAM; 7 Internal Medicine, Keystone Health, Chambersburg, USA

**Keywords:** sleeping sickness, melarsoprol, nifurtimox, eflornithine, fexinidazole, trypanosoma brucei

## Abstract

Human African trypanosomiasis (HAT), or sleeping sickness disease, is an infection caused mainly by *Trypanosoma brucei gambiense-*human African trypanosomiasis (g-HAT) and is transmitted by tsetse flies. The disease goes through two stages: hemolymphatic and meningo-encephalic phases. The treatment for the second stage has changed from melarsoprol or eflornithine to nifurtimox-eflornithine combination therapy (NECT) and fexinidazole. We aimed to systematically review the literature on the efficacy and toxicity of fexinidazole and NECT. We used PubMed advanced strategy and Google Scholar databases, including clinical trials and observational studies on humans in the last 20 years in the English literature. Applying the inclusion/exclusion criteria, we reviewed eight studies. We used Preferred Reporting Items for Systematic Reviews and Meta-Analysis (PRISMA) and Meta-analysis of Observational Studies in Epidemiology (MOOSE) protocol. For assessing bias, we used the Cochrane Collaboration’s tool for risk assessment of the clinical trials and the Robins-I tool for the observational studies. Overall, the clinical trials showed that NECT was non-inferior to eflornithine. The proportion of patients discharged alive is higher in patients treated with NECT vs. patients treated with eflornithine. Gastrointestinal complaints are a common side effect of NECT therapy, while fearful but relatively rare convulsions can also occur. The main limitation among the studies of NECT was the lack of blinding because most of them were open-label. Fexinidazole, the new oral medication showed is effective and safe for the treatment of g-HAT infection. Because of their convenience, fexinidazole is preferred over NECT therapy, oral vs. IV infusion in the first and second stages of the disease. Compared to older therapies, fexinidazole and NECT are more effective and safer than eflornithine and melarsoprol monotherapy.

## Introduction and background

Human African trypanosomiasis (HAT), also called sleeping sickness, is a tropical disease that affects sub-Saharan African countries [1]. Annually, about 12,000 cases of sleeping sickness are reported; however, other reports suggest that the actual case count could be five times higher [2]. HAT is a protozoan parasitic infection that is classically transmitted by the blood-sucking tsetse flies by mainly three species of trypanosomes: *Trypanosoma vivax*, *Trypanosoma congolense*, and *Trypanosoma gambiense* [3]. The disease goes through two stages: the first is the hemolymphatic phase, which is effectively treated with pentamidine, and the second is the meningo-encephalitic stage. The second stage is characterized by parasitic invasion of the central nervous system after crossing the blood-brain barrier, which leads to psychiatric and neurological disorders, coma, and/or death [1,4]. Drugs to treat second-stage HAT are highly toxic, old, and encounter parasite resistance [5,6]. Three drugs are used to treat second phase sleeping sickness: melarsoprol, eflornithine, and nifurtimox [5]. Since 1942, the primary drug of treatment for the second stage was melarsoprol, which is an arsenic derivative with high toxicity, which caused life-threatening side-effects, in particular reactive encephalopathy that develops within four days of the start of therapy and affects 5-10% of treated patients. The mortality of melarsoprol-induced reactive encephalopathy was 50% [7]. Eflornithine, used since 1990, was better tolerated than melarsoprol. However, the drug is difficult to administer, requiring 56 infusions, four daily slow infusions for 14 days [2]. In 2009, the WHO Essential Medicine List (WHO EML) included nifurtimox-eflornithine combination therapy (NECT) as the first-line treatment for the second stage, with high cure rates (95-98%) and lower fatality rates (<1%) [1]. The first pilot three-therapy trial (TTT) was conducted in Uganda in 2001 and was terminated early because of high toxicity in the melarsoprol group [2]. A second large-scale NECT trial was conducted in the Republic of Congo, and this led to the inclusion of NECT in the WHO EML [4]. The most recently studied medication is fexinidazole. The first in-human clinical trials involving fexinidazole began in 2009 and the phase II/III clinical trials of fexinidazole started years after in 2012. Further clinical trials demonstrated that fexinidazole treatment was well tolerated with an acceptable safety profile [1].

According to the WHO, HAT is a neglected tropical disease and it is vital to continue investigations on this disease [8]. We aimed to conduct a systematic review of the clinical trials and observational studies to investigate the efficacy and toxicity of fexinidazole and NECT on patients in the second stage of sleeping sickness. The purpose of this study is to consolidate the knowledge of the treatment of sleeping sickness with fexinidazole and NECT and to compare these studies with older medications, such as melarsoprol and eflornithine.

## Review

Materials and methods

For this systematic review, we used the Preferred Reporting Items for Systematic Reviews and Meta-Analysis (PRISMA). We also used the Meta-analysis Of Observational Studies in Epidemiology (MOOSE) protocol for observational studies.

Eligibility Criteria and Study Selection

We only included clinical trials and observational studies on humans in the last 20 years in the English literature. We excluded studies other than clinical trials and observational studies because they give a higher degree of evidence. Also, we excluded papers that did not fulfill the aims of our study. After screening the studies, we included papers with the following criteria: (1) patients - individuals with African trypanosomiasis; (2) intervention - use of fexinidazole or nifurtimox + eflornithine in patients with African trypanosomiasis; (3) comparator - placebo, control group, or standard treatment; (4) outcomes - survival rate, drug toxicity

Database and Search Strategy

A review of the literature using PubMed and Google Scholar databases was performed during May 2021 to July 2021. The search terms used were "African trypanosomiasis"[Title/Abstract] AND "nifurtimox eflornithine"[Title/Abstract] OR "sleeping sickness"[Title/Abstract] AND "nifurtimox eflornithine"[Title/Abstract] OR "African trypanosomiasis"[Title/Abstract] AND "Fexinidazole"[Title/Abstract] OR "sleeping sickness"[Title/Abstract] AND "Fexinidazole"[Title/Abstract].

Data Extraction and Analysis

We collected the following information for each study: the first author's last name, year of publication, country, study type, study design, number of patients in the treatment group, number of patients in the control group, and outcomes.

Bias Assessment

For assessing bias, we use the Cochrane Collaboration’s tool for risk assessment of the clinical trials [9] and the Robins I tool for the observational studies [10].

Results

**Figure 1:**
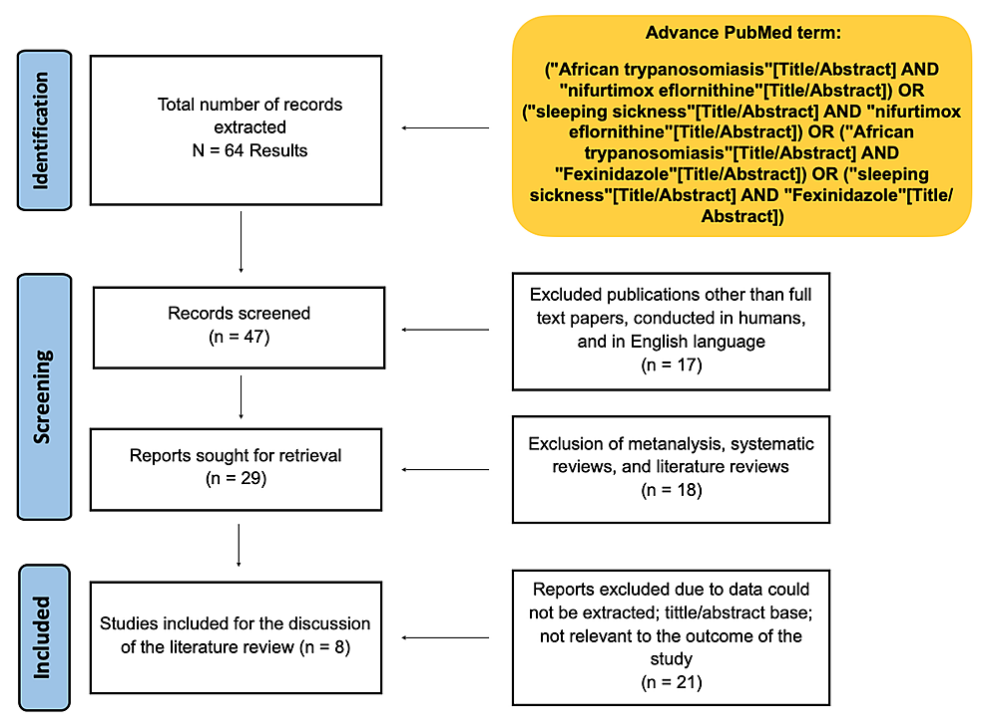
Results of the study using the PRISMA flow chart. PRISMA: Preferred Reporting Items for Systematic Reviews and Meta-Analysis

Figure 1 below shows the results of the study using the PRISMA flow chart.

Table 1 below shows the methodology and main outcomes of the studies selected for the systematic review [2,4,11-16]. 

**Table 1:** Results of the observation studies and clinical trials of the systematic review. ITT: intention-to-treat, PP: per protocol, AE: adverse event, NECT: nifurtimox-eflornithine combination therapy, SAE: serious adverse events, HAT: human African trypanosomiasis, g-HAT: *Trypanosoma brucei gambiense-*human African trypanosomiasis

Author, year of publication, country	Study type	Study design	Number of patients in treatment group	Number of patients control group	Outcomes
Priotto et al., 2007, Republic of Congo [11]	Randomized controlled clinical trial, open label	Patients were randomly selected to receive either eflornithine or eflornithine + nifurtimox. Patients were followed for 18 months. One hundred and three (103) patients in the second stage were enrolled in the study.	52	51	The cure rate for eflornithine was 94.1% and for eflornithine + nifurtimox was 96.3% Severe reaction was: eflornithine 25.5% and eflornithine + nifurtimox 9.6%
Kansiime et al., 2018, Uganda [4]	Multicenter, open label, randomized, non-inferiority clinical trial	Patients were selected to receive NECT or the standard eflornithine regimen. They were followed for 18 months post-treatment, and primary endpoint was the cure rate, determined as the proportion of patients alive and without laboratory signs of infection.	55	54	One hundred and nine (109) were enrolled, and they contributed to ITT and 105 to PP. The Cure rate for NECT was 90.9% and 88.9 for eflornithine alone in ITT populations. The same was 90.6 and 88.5%, respectively in the PP population.
Schmid et al., 2012, Democratic Republic of Congo [12]	Multicenter, open label, single arm, phase IIIb study	All the patients were treated with NECT to assess the response for of 2nd stage of *Trypanosoma brucei gambiense* HAT. The primary outcome was the proportion of patients discharged alive from the hospital, and the second outcome was safety based on treatment-emergent adverse events (AEs) occurring during hospitalization. The patients were followed for 13 months.	629	0	Proportion of patients who were discharged alive after treatment completion was 98.4% (619/629; 95% CI [97.1%; 99.1%]). Of the ten patients who died during hospitalization, eight presented in a bad health condition at baseline; one death was assessed as unlikely related to treatment. Most common AEs were gastrointestinal (61%), general (46%), nervous system (mostly central; 34%) and metabolic disorders (26%).
Franco et al., 2012, Africa [13]	Observational study	Patients were selected for assessing the safety and efficacy of NECT in routine use. Safety was assessed from the characterization of AE during treatment, and efficacy was assessed from the register of relapses in patients during the two years following treatment.	1735	0	At least one AE was described in 1043 patients (60.1%), and a total of 3060 AE were reported. SAE were reported for 19 patients (1.1% of treated), leading to nine deaths (case fatality rate of 0.5%)
Alirol et al., 2012, Democratic Republic of Congo [14]	Retrospective cohort study	The study included 684 second-stage HAT patients (including 120 children) treated with NECT. All second-stage HAT patients treated with NECT between 1 January 2010 and 30 June 2011 were included in the analysis. Adverse effects were recorded and graded.	684	0	Among patients treated with NECT, 86% experienced at least one adverse effect during treatment. On average, children experienced fewer AEs than adults. In comparison with previous treatments, NECT was effective, safe, and well-tolerated in non-trial settings in DRC, further supporting the roll-out of NECT as first-line treatment in second-stage *Trypanosoma brucei gambiense* HAT. Tolerance was good in children.
Priotto et al., 2009, Republic of the Congo and the Democratic Republic of the Congo [2]	Multicenter, randomized, phase III, non-inferiority trial, open-label	Patients aged 15 years or older with confirmed second-stage *Trypanosoma brucei gambiense* infection were randomly assigned by computer-generated randomization sequence to receive intravenous eflornithine for 7 days with oral nifurtimox for 10 days or intravenous eflornithine for 14 days. They were followed for 18 months.	143	144	131 (91.6%) of 143 patients assigned to eflornithine and 138 (96.5%) of 143 patients assigned to NECT were cured at 18 months. Drug-related adverse events were frequent in both groups; 41 (28.7%) patients in the eflornithine group and 20 (14.0%) in the NECT group had major (grade 3 or 4) reactions. The efficacy of NECT is non-inferior compared to eflornithine monotherapy
Mesu et al., 2018, Democratic Republic of the Congo [15]	Randomized, phase 2/3, open-label, non-inferiority trial	394 patients were randomly assigned, 264 to receive fexinidazole and 130 to receive NECT. Success at 18 months was recorded in 91% of patients given fexinidazole and 98% of patients given NECT.	264	130	Success rates at 18 months were higher than expected in both treatment groups: 91.2% in the fexinidazole group (89% expected) and 97.6% in the NECT group (94% expected). The most frequently reported adverse events were: headache, vomiting, nausea, and insomnia; the latter being the largest difference between groups (28% vs. 12%). Oral fexinidazole is effective and safe for the treatment of *Trypanosoma brucei gambiense* infection compared with NECT in late-stage HAT patients.
Mesu et al., 2021, Democratic Republic of the Congo [16]	Prospective, multicentre, open-label, single-arm cohort study	Patients were classified into stage 1 or early stage 2 g-HAT groups following evidence of trypanosomes. Study participants were followed up on day 5 and day 8 during treatment, at end of treatment on day 11, at end of hospitalization on days 11-18, at week 9 for a subset of patients, and after 6 months, 12 months, and 18 months. The primary endpoint was treatment success at 12 months.	230	0	Treatment was effective at 12 months for 99% of patients (95% CI 96.2-99.7): 98% of patients (95.4-99.7) with stage 1 and 100% of patients (91.4-100.0) with early stage 2. The most frequent adverse events were headache and vomiting.

Study Limitations

Schmid et al. argued that it was confusing to differentiate if the symptoms were part of the disease or a side effect of the medication in the study. Additionally, there was background malnutrition in some patients, which could mask other symptoms. Finally, the study was open-label, increasing the risk of observer and performance bias [12].

Franco et al. and Alirol et al.’s studies were limited to the pharmacovigilance data and the subjective component in the report group as adverse events versus severe adverse events. This systematic error increased the risk of type 1 errors [13,14].

The study by Kansiime et al. was limited because it had a small sample size which was attributed to loss of follow-up of patients and distribution of medications to the sample population. This small dataset increased the chances of type II errors and affected the validity of the study [4].

Studies by Kansiime et al. and Priotto et al. were limited due to the hospitalization duration for the two groups because they had different treatment schedules. A longer hospitalization time means a long period of observation and recording of more side effects [2,4].

The studies by Schmid et al., Kansiime et al., and Priotto et al. were limited by their study design as open-labeled, which was unavoidable as a blinded design allows different modes of administration of eflornithine monotherapy and NECT to be given. In these studies, the timing, choice, and mode of administration of treatments differed from sample sites. This lack of homogeneity is a consequence of resource-stricken areas from which the sample population was chosen. This heterogenicity posed a restraint on possible future reproducibility and generalizations of the study [2,4,12-14].

The study conducted by Mesu et al. in 2018 presented an attrition bias because the group that received fexinidazole had 18 premature withdraws, and the group that received NECT had five withdrawals. Moreover, open-label studies increase the high risk of detection bias and performance bias [15].

**Figure 2:**
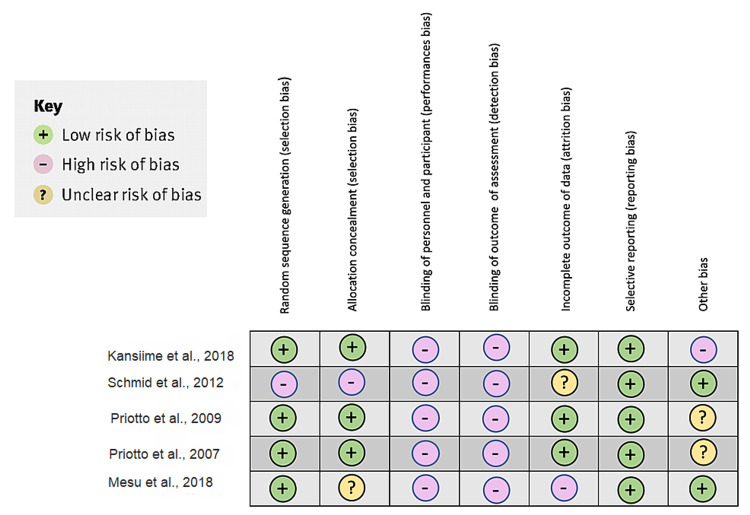
Bias analysis of the clinical trials for the systematic review of the study

Another study conducted by Mesu et al. in 2021 was limited because it had a single treatment group, and was an open-label study design [16]. As it is a rare disease, patients available to take part in the trial were limited. In addition, the different modes of treatment administration would have made a double-blind comparative study impossible [14]. Figure 2 shows the bias analysis of the clinical trials in the systematic review [2,4,11,12,15].

Table 2 shows the bias analysis of the observation studies the systematic review of the study [13,14,16].

**Table 2:** Bias analysis of the observation studies the systematic review of the study

Study	Confounding	Selection bias	Classification of intervention	Deviation from intervention	Missing data	Measurement of the outcome	Selection of reported result
Franco et al., 2012 [13]	Low risk	Moderate risk	Low risk	Moderate risk	Moderate risk	Low risk	Low risk
Alirol et al., 2013 [14]	Low risk	Low risk	Low risk	Moderate risk	Moderate risk	Low risk	Low risk
Mesu et al., 2021 [16]	Low risk	Low risk	Moderate risk	Moderate risk	Moderate risk	Moderate risk	Low risk

Discussion

We analyzed the efficacy and toxicity of fexinidazole and NECT and compared them to previous treatments. We will discuss our findings and provide new directions in the treatment of sleeping sickness. 

Efficacy of Nifurtimox + Eflornithine

The first drug used to treat second-stage HAT was melarsoprol which had 94% of effectiveness [17]. Besides the high levels of toxicity, melarsoprol has developed resistance by gene modifications in the aquaglyceroporin 2 (AQP2) gene [8]. The increased resistance to the drug prompted the scientific community to search for new treatments.

Three clinical trial studies showed that the efficacy of NECT is non-inferior to standard eflornithine monotherapy [3,5,7]. In the clinical trial by Schmid et al., the primary outcome was the proportion of patients discharged alive from the hospital [12]. The study was the first to include special populations that were not included in previous clinical trials, such as children below 15 years old and pregnant women [12]. In-hospital safety of the drug was comparable to previous studies [10].

Three clinical trials defined cure rate as the absence of trypanosomes in the body fluids and leucocyte count less than 20 cells per μL 18 months after treatment [3,5,7]. While the study by Schmid et al., the primary outcome was the proportion of patients discharged from the hospital [12].

The administration of NECT is easier and requires fewer hospital resources than eflornithine monotherapy [12]. Eflornithine requires four daily slow infusions for 14 days, a total of 56 infusions, which makes them inconvenient for most patients [2]. Meanwhile, NECT requires only 14 slow infusions administered every 12 hours for one week and oral treatment with nifurtimox for 10 days [12].

Efficacy of Fexinidazole

Fexinidazole is a new oral drug that has been used to treat first-stage and advanced-stage sleeping sickness in patients that live in remote areas with few health resources. Fexinidazole is a 5-nitroimidazole drug that has two principal metabolites, sulfoxide and sulfone, that kill a range of *T. brucei* parasite strains [18]. A trial in Africa showed that fexinidazole was less effective in patients with severe disease than NECT, which had a 98% rate of success. However, fexinidazole is well tolerated and is easier to use (take by mouth) than NECT that requires multiple infusions [15]. Mesu et al. 2018 conducted a randomized study where 264 patients received fexinidazole, and 130 patients received NECT. They found that fexinidazole was non-inferior to NECT, and there was no difference in the proportion of patients that experience treatment-related adverse events (215 {81%} in the fexinidazole group vs. 102 {79%} in the NECT group), showing that oral fexinidazole is effective and safe [15].

In the latest fexinidazole clinical trial by Mesu et al. in 2021, they assessed the safety and efficacy of fexinidazole in early *Trypanosoma brucei gambiense*-human African trypanosomiasis (g-HAT). A total of 189 patients with stage 1 g-HAT and 41 patients with early-stage 2 g-HAT were included, which completed a 10 day treatment period. Study participants were followed up until the end of hospitalization, and after six months, 12 months, and 18 months [18]. Due to the small proportion of patients with early-stage 2 g-HAT (41 patients), the objective was only reached for patients with stage 1 g-HAT. Headache and vomiting were the most frequently reported adverse events overall, however, the majority were mild or moderate and none led to treatment discontinuation. Thus, fexinidazole's benefit-risk balance is clearly positive. With an overall treatment success of 99% at 12 months and 98% at 18 months, the present study shows that fexinidazole benefits adult and adolescent patients with stage 1 and early stage 2 g-HAT, and consolidates the role of oral fexinidazole as a valuable first-line treatment option in early g-HAT. In conclusion, fexinidazole is safe and effective at killing the parasite in both acute and chronic models. Fexinidazole might contribute to achieving the WHO target of eliminating* Trypanosoma brucei gambiense* by 2030 [16].

Toxicity in the Treatment of Sleeping Sickness

The resistance to melarsoprol and inconvenience with the use of eflornithine was not the only problem. Table 3 shows the mechanisms of action and toxicity of melarsoprol and eflornithine [8-12]. 

**Table 3:** Mechanism of action and toxicity of melarsoprol and eflornithine.

	Melarsoprol	Eflornithine
Mechanism of action	Prodrug, metabolized to melarsen oxide, and irreversibly binds to sulfhydryl groups on pyruvate kinase, and disrupts energy production in the parasite, preventing trophozoite multiplication	Irreversible ornithine decarboxylase inhibitor
Toxicity	Reactive encephalitis, agranulocytosis, peripheral neuropathy, cardiac arrhythmias, and hypertension	Pancytopenia, diarrhea, hallucinations, convulsions, attacks the immune system

One of the most worrisome side effects was reactive encephalitis which occurred in 5-10% of patients, 10 days after treatment due to rapid lysis of the trypanosomes [8].

Nifurtimox is an orally administered drug used to treat American trypanosomiasis (Chagas disease). Nevertheless, nifurtimox has also been used to treat African trypanosomiasis. Nifurtimox, a nitrofurans derivate, forms a nitro-anion radical metabolite that reacts with nucleic acids of the parasite and inhibits a parasite-specific antioxidant defense enzyme called trypanothione reductase, causing DNA breakdown. Also, it creates oxygen-derived free radicals, and the accumulation of cytotoxic levels results in parasite death [19]. Some studies showed that 50% of patients that received nifurtimox relapsed, but when it was combined with melarsoprol, there was not a relapse. However, this combination was highly toxic. Combination therapy with eflornithine + nifurtimox was safer and easier to administer than treatment with eflornithine alone [20]. NECT might also prevent or delay the emergence of drug-resistant organisms.

Two observational studies by Alirol et al. and Franco et al. recorded the toxicity of NECT in the treatment of second-stage trypanosomiasis [13,14]. The total data collected were from 2419 patients (382 children). Both studies showed that NECT has a relatively good safety profile. The most frequent adverse effect in both databases included: vomiting, nausea, headache, abdominal pain, and musculoskeletal pain. Adults had a four-fold increased chance to develop an adverse event than children, and this could be that nifurtimox has a better safety profile in children, or because children had a better general condition on admission [13]. The mortality rate was low in both studies (0.15%).

Regarding drug toxicity, patients in the NECT group reported at least one adverse event compared to the eflornithine group [11]. Nevertheless, NECT was associated with a lower frequency of infections, fever, hypertension, neutropenia, diarrhea, dysphagia, and showed fewer serious adverse events (coma, seizures, ataxia, paralysis, encephalopathy) and deaths compared to eflornithine monotherapy [11,13]. The most frequent adverse event reported in the studies during NECT were gastrointestinal disorders, followed by neuropsychiatric disorders. The most common were abdominal pain, headache, and vomiting. The reduced bone marrow toxicity was reflected by the lower frequency of neutropenia and anemia in the NECT group compared to the eflornithine monotherapy group. This difference could be explained by the varying dosage duration of eflornithine in the two groups (control 14 days vs. NECT seven days). Also, there was a lower frequency of infection in the NECT group, as fewer infusions were needed, thus reducing catheter-related infections (56 vs. 14) [2].

Two clinical trials found fexinidazole to be a well-tolerated drug, particularly in patients with stage 1 disease. Pooled analysis of safety data suggested an acceptable safety profile for fexinidazole. The most common adverse events were mild or moderate (vomiting, nausea, asthenia, decreased appetite, headache, insomnia, tremor, and dizziness) and only four serious adverse events were considered as possibly related to fexinidazole were reported (two reports of personality change, one of acute psychosis and one of hyponatremia). Several of the most frequently observed treatment-emergent adverse events occurred more commonly in the fexinidazole group than in the NECT group [15,16].

## Conclusions

Three clinical trial studies showed that the efficacy of NECT is non-inferior to eflornithine monotherapy, and the proportion of patients discharged alive from the hospital was high. The administration of NECT is easier and requires fewer hospital resources than eflornithine monotherapy. The two observational studies demonstrated that NECT has a good safety profile. Even if most patients reported at least one adverse event, this was mild with no complications, compared to eflornithine monotherapy and melarsoprol. Fexinidazole is the only available oral monotherapy regimen developed and tested so far to treat patients with g-HAT. From a clinical practice perspective, a positive effect on patient management is expected with the use of this oral regimen with acceptable efficacy compared with the NECT standard of care. This is a great advantage and much easier and is not associated with any of the potential hospitalization complications, meaning that these patients could receive home-based treatment. The availability of an oral regimen should also have positive financial effects both at the patient level and at the healthcare level, because oral administration requires fewer medical resources, alleviating the financial burden on HAT control programs.

## References

[1] Neau P, Hänel H, Lameyre V, Strub-Wourgaft N, Kuykens L (2020). Innovative partnerships for the elimination of human African trypanosomiasis and the development of fexinidazole. Trop Med Infect Dis.

[2] Priotto G, Kasparian S, Mutombo W (2009). Nifurtimox-eflornithine combination therapy for second-stage African Trypanosoma brucei gambiense trypanosomiasis: a multicentre, randomised, phase III, non-inferiority trial. Lancet.

[3] Shereni W, Neves L, Argilés R, Nyakupinda L, Cecchi G (2021). An atlas of tsetse and animal African trypanosomiasis in Zimbabwe. Parasit Vectors.

[4] Kansiime F, Adibaku S, Wamboga C (2018). A multicentre, randomised, non-inferiority clinical trial comparing a nifurtimox-eflornithine combination to standard eflornithine monotherapy for late stage Trypanosoma brucei gambiense human African trypanosomiasis in Uganda. Parasit Vectors.

[5] Legros D, Ollivier G, Gastellu-Etchegorry M, Paquet C, Burri C, Jannin J, Büscher P (2002). Treatment of human African trypanosomiasis - present situation and needs for research and development. Lancet Infect Dis.

[6] Kennedy PG (2013). Clinical features, diagnosis, and treatment of human African trypanosomiasis (sleeping sickness). Lancet Neurol.

[7] Seixas J, Atouguia J, Josenando T, Vatunga G, Bilenge CM, Lutumba P, Burri C (2020). Clinical study on the melarsoprol-related encephalopathic syndrome: risk factors and HLA association. Trop Med Infect Dis.

[8] Fairlamb AH, Horn D (2018). Melarsoprol resistance in African trypanosomiasis. Trends Parasitol.

[9] Higgins JP, Altman DG, Gøtzsche PC (2011). The Cochrane Collaboration's tool for assessing risk of bias in randomised trials. BMJ.

[10] Sterne JA, Hernán MA, Reeves BC (2016). ROBINS-I: a tool for assessing risk of bias in non-randomised studies of interventions. BMJ.

[11] Priotto G, Kasparian S, Ngouama D, Ghorashian S, Arnold U, Ghabri S, Karunakara U (2007). Nifurtimox-eflornithine combination therapy for second-stage Trypanosoma brucei gambiense sleeping sickness: a randomized clinical trial in Congo. Clin Infect Dis.

[12] Schmid C, Kuemmerle A, Blum J (2012). In-hospital safety in field conditions of nifurtimox eflornithine combination therapy (NECT) for T. b. gambiense sleeping sickness. PLoS Negl Trop Dis.

[13] Franco JR, Simarro PP, Diarra A, Ruiz-Postigo JA, Samo M, Jannin JG (2012). Monitoring the use of nifurtimox-eflornithine combination therapy (NECT) in the treatment of second stage gambiense human African trypanosomiasis. Res Rep Trop Med.

[14] Alirol E, Schrumpf D, Heradi JA, Riedel A, de Patoul C, Quere M, Chappuis F (2013). Nifurtimox-eflornithine combination therapy for second-stage gambiense human African trypanosomiasis: médecins sans frontières experience in the Democratic Republic of the Congo. Clin Infect Dis.

[15] Mesu VK, Kalonji WM, Bardonneau C (2018). Oral fexinidazole for late-stage African Trypanosoma brucei gambiense trypanosomiasis: a pivotal multicentre, randomised, non-inferiority trial. Lancet.

[16] Mesu VK, Kalonji WM, Bardonneau C (2021). Oral fexinidazole for stage 1 or early stage 2 African Trypanosoma brucei gambiense trypanosomiasis: a prospective, multicentre, open-label, cohort study. Lancet Glob Health.

[17] Lutje V, Seixas J, Kennedy A (2013). Chemotherapy for second-stage human African trypanosomiasis. Cochrane Database Syst Rev.

[18] Kaiser M, Bray MA, Cal M, Trunz BB, Torreele E, Brun R (2011). Antitrypanosomal activity of fexinidazole, a new oral nitroimidazole drug candidate for treatment of sleeping sickness. Antimicrob Agents Chemother.

[19] Pund S, Joshi A (2017). Nanoarchitectures for neglected tropical protozoal diseases: challenges and state of the art. Nano- and Microscale Drug Delivery Systems: Design and Fabrication.

[20] Checchi F, Piola P, Ayikoru H, Thomas F, Legros D, Priotto G (2007). Nifurtimox plus eflornithine for late-stage sleeping sickness in Uganda: a case series. PLoS Negl Trop Dis.

